# Gestational diabetes mellitus subtypes: from one-size-fits-all to precision medicine

**DOI:** 10.1007/s42000-025-00700-z

**Published:** 2025-08-08

**Authors:** Kleoniki I. Athanasiadou, Stavroula A. Paschou

**Affiliations:** Endocrine Unit and Diabetes Centre, Department of Clinical Therapeutics, Alexandra Hospital, School of Medicine, National and Kapodistrian University of Athens, Athens, Greece

Gestational diabetes mellitus (GDM) is a common complication of pregnancy associated with multiple adverse perinatal outcomes. GDM incidence varies widely, with a global average prevalence of 14% (ranging between 7% and 28%) according to the screening method -universal or “risk factor-based”- and the applied diagnostic criteria [1, 2]. The International Association of Diabetes in Pregnancy Study Group (IADPSG) expanded the diagnostic criteria for GDM based on the findings of the Hyperglycaemia and Adverse Pregnancy Outcomes (HAPO) study, thus broadening the spectrum of women being diagnosed with GDM. The HAPO study demonstrated that pregnant women with mild hyperglycaemia, below the previous diagnostic thresholds, presented an increased risk of perinatal complications (i.e., macrosomia, large for gestational age (LGA), fetal hyperinsulinaemia, neonatal hypoglycaemia, primary caesarean delivery, pre-eclampsia, shoulder dystocia, neonatal intensive care unit (NICU) admission) [3].

The worldwide adoption of uniform diagnostic criteria for GDM, though, and the universal screening of all pregnant women for GDM are still matters of considerable controversy. Additionally, another unclear issue, which the latest research has tried to clarify is whether all women with GDM face the same perinatal risks. ’For years, GDM was considered a relatively homogenous clinical condition and the oral glucose tolerance test (OGTT) was used to confirm or exclude the presence of GDM. All women were treated similarly, as having a transient hyperglycaemic disorder of pregnancy, and were managed with the same well-defined treatment plan, including diet/medical nutrition therapy, physical activity, and glucose-lowering medications (metformin or insulin therapy) [4]. However, impressive research findings, analysing metabolic and clinical parameters, have recently shown that GDM, like type 2 diabetes, is a heterogeneous condition. The presence of different GDM subtypes, sharing diverse pathophysiological procedures, indicates the potential for introducing precision medicine in GDM care [4, 5]. From a clinical perspective, the GDM subtypes can be classified using combinations of abnormal OGTT values of pregnant women with GDM, as they have been associated with different pathophysiological mechanisms and metabolic phenotypes.

Fasting hyperglycaemia represents an impairment in insulin secretion, while post-load hyperglycaemia represents a defect in insulin sensitivity [6]. Women with fasting, post-load, or combined hyperglycaemia present variability in perinatal outcomes, including macrosomia, caesarean section, hypertensive disorders of pregnancy, prematurity, and need for glucose-lowering medications. Generally, women with hyperglycaemia at one time point share milder forms of GDM. It has been shown that women with isolated post-load hyperglycaemia present the most favourable perinatal outcomes compared to women of another GDM subtype. The recognition of different GDM subtypes determined by the OGTT has established the presence of heterogeneity in GDM. An impressive finding by White et al. showed that women with GDM and isolated post-load hyperglycaemia had similar insulin sensitivity and insulin secretion with normoglycaemic women, indicated by the calculated HOMA indices [7]. This finding suggests that the subtype of isolated post-load hyperglycaemia may be the mildest GDM form. Further research on the underlying pathophysiological mechanisms is required to confirm this hypothesis. Multiple relevant studies conclude that women with concomitant fasting and post-load hyperglycaemia share the most severe metabolic phenotype, in terms of insulin sensitivity, insulin secretion, and adverse perinatal outcomes, followed by women with isolated fasting hyperglycaemia [8–10]. Apart from the clinical variability in pregnancy outcomes, biochemical, lipidomic, metabolomic, and genetic differences have also been observed among the GDM subtypes. The heterogeneity in fetal response to maternal hyperglycaemia could possibly be attributed to genetic variations affecting the feto-maternal unit [11].

In other words, the classification of women with GDM according to the underlying pathophysiological mechanisms involving dysfunctions in insulin sensitivity and insulin resistance has made considerable progress towards offering individualised treatment and enhances our understanding of potential pregnancy complications and outcomes. Recent studies have identified a number of clinical factors that can be used as precision markers for risk-stratification of women with GDM [12]. Previous history of GDM, increased body mass index (BMI) > 30 kg/m^2^, increased HbA1c level (within the prediabetic range), increased fasting plasma glucose level on the OGTT (> 100 mg/dl), advanced maternal age (≥ 35 years old), and family history of diabetes have been associated with an increased risk of requiring glucose-lowering medications rather than diet al.one and unfavourable pregnancy outcomes [13]. Obviously, the presence of these clinical variables indicates a high-risk GDM subtype.

The introduction of technology into diabetes care has transformed current clinical practice. In pregnancy, the increasing use of continuous glucose monitoring (CGM) using wearable sensors has significantly improved the quality of life of pregnant women eliminating the need for multiple finger-prick tests during the day. Another advantage is the potential for remote monitoring of blood glucose levels by clinicians (e.g. the GDm-Health platform in the UK) followed by virtual consultations (tele-medicine), if needed, reducing the need for physical appointments. Projecting into the future, the development of official predictive models using identified precision markers and advanced models, such as data-driven cluster analysis as proposed by Salvatori et al., represents the next step in integrating technology to GDM care [12]. Another recent study analysed placental gene expression to detect differences between GDM A1 (diet-controlled) and A2 (pharmacological management) subtypes [14]. The establishment of such models in clinical practice would require future prospective studies to validate these tools and assess cost-effectiveness and their impact on pregnancy and long-term maternal outcomes.

Another important aspect of precision medicine in GDM pertains to the increased risk for type 2 diabetes and metabolic complications that these women will possibly face in later life, highlighting the significance of universal screening for GDM. A recent meta-analysis demonstrated that women with a history of GDM have a nearly ten-fold risk of developing type 2 diabetes compared to normoglycaemic women [15, 16]. In view of this unfavourable perspective, it is important to ensure that women with a history of GDM will complete their postnatal glucose screening with a postnatal OGTT within 2 to 3 months after delivery and every 1 to 3 years thereafter [17, 18]. Almost all guidelines align with this screening pattern, the aim being to identify as many women with type 2 diabetes or prediabetes as possible. In the UK, on the other hand, the NICE-2015 guidance supports the measurement of fasting plasma glucose levels after 6 weeks or HbA1c levels 13 weeks postpartum, this policy being based on cost-effectiveness [19]. Despite official recommendations, the postpartum glucose screening rates remain to date low. This has been attributed to several factors. For instance, the increased responsibilities of the new mother are undoubtedly a burden; however, it has also been noted that postpartum glucose screening guidance is sometimes not provided at discharge from the obstetric unit. Raising awareness regarding future metabolic risks and providing clear guidance for postpartum testing to new mothers at discharge could improve adherence.

In conclusion, GDM is a heterogenous condition that requires an individualised treatment strategy, rather than the current ‘’one-size-fits-all’’ approach. The various levels of insulin resistance and impaired insulin secretion are reflected in the OGTT glucose levels at the different time points. The use of this clinical tool can be helpful for risk stratification of women with GDM, identifying those with milder or more severe forms of hyperglycaemia. The integration of prognostic models evaluating the risk of GDM development in all pregnant women in clinical practice holds promise for increasing the detection of women at high risk early in pregnancy while simultaneously aiming for close monitoring utilising diabetes technology features, and early intervention to improve maternal and fetal outcomes. Lifestyle interventions and postpartum glucose screening, especially in women with high-risk GDM subtypes, can have a significant impact on overall health promotion thereby delaying or preventing type 2 diabetes development.
